# Differential Expression Of Adhesion Molecules Within The Human Thymus

**DOI:** 10.1155/1994/49301

**Published:** 1994

**Authors:** Julie Naima Reza, Mary A. Ritter

**Affiliations:** Department of Immunology Royal Postgraduate Medical School Du Cane Road London W12 ONN United Kingdom

**Keywords:** Adhesion molecules, macrophages, stromal cells, thymus

## Abstract

Development of a diverse, MHC-restricted yet self-tolerant T-cell repertoire occurs within the thymus, and requires contact between developing T cells and their stromal microenvironment. Such interactions are likely to depend on the combinatorial effect of specific adhesion molecules. As a preliminary step to determining their role in T-cell development, we have studied the distribution of LFA-1/ICAM-1, CD2/LFA-3, VLA-4/VCAM-1, and HECA 452-antigen/E-Selectin ligand pairs on frozen sections of human thymus. Using two color-immunohistochemistry, and a variety of cell-lineage markers that reveal the nature of the cells on which these adhesion molecules are located, we find a differential distribution of adhesion molecules, with some being shared by both endothelial and epithelial cells. We also identify the VCAM-1-positive subpopulation as cortical macrophages. The relevance of these findings to thymopoiesis is discussed.


*Developmental Immunology*, 1994, Vol 4, pp. 55–64
Reprints available directly from the publisher
Photocopying permitted by license only

© 1994 Harwood Academic Publishers GmbH
Printed in Singapore

### Differential Expression of Adhesion Molecules within the Human Thymus

JULIE NAIMA REZA* and MARY A. RITTER

*Department of Immunology, Royal Postgraduate Medical School, Du Cane Road, London W12 ONN, U.K.*

Development of a diverse, MHC-restricted yet self-tolerant T-cell repertoire occurs within the thymus, and requires contact between developing T cells and their stromal microenvironment. Such interactions are likely to depend on the combinatorial effect of specific adhesion molecules. As a preliminary step to determining their role in T-cell development, we have studied the distribution of LFA-1/ICAM-1, CD2/LFA-3, VLA-4/VCAM-1, and HECA 452-antigen/E-Selectin ligand pairs on frozen sections of human thymus. Using two color-immunohistochemistry, and a variety of cell-lineage markers that reveal the nature of the cells on which these adhesion molecules are located, we find a differential distribution of adhesion molecules, with some being shared by both endothelial and epithelial cells. We also identify the VCAM-1–positive subpopulation as cortical macrophages. The relevance of these findings to thymopoiesis is discussed.

KEYWORDS: Adhesion molecules, macrophages, stromal cells, thymus.

### INTRODUCTION

A fully functional immune system requires the generation of a diverse T-cell repertoire, which is both major histocompatibility–(MHC) restricted and self-tolerant. This development occurs in the thymus (reviewed by Boyd and Hugo, 1991; Ritter and Crispe, 1992; Boyd et al., 1993; and Ritter and Boyd, 1993), where bone marrow-derived pluripotent stem cells first enter the thymus and subsequently interact with stromal cells. The developing thymocytes change phenotypes and also undergo positive and negative selection, rendering them self-MHC-restricted (Sprent et al., 1975; Jenkinson et al., 1985; Kappler et al., 1987; Marrack et al., 1988) and self-tolerant (Lo and Sprent, 1986; Marrack et al., 1988; Berg et al., 1989), respectively. Thus, interactions between thymocytes and stromal cells, whether cell-cell or via soluble molecules, must provide signals for the migration, proliferation, maturation, and selection of T cells, as well as signals for maintaining the structural integrity of the thymic microenvironment. Details about such interactions are unclear, but it is likely that adhesion molecules are involved.

Adhesion molecules can be divided into several groups (reviewed by Albelda and Buck, 1990; Springer, 1990; and Pigott and Power, 1993): (a) the Immunoglobulin superfamily (e.g., CD2, Vascular Cell Adhesion Molecule [VCAM]-1, Intercellular Cell Adhesion Molecule [ICAM]-1, and Leucocyte Function Related Antigen [LFA]-3), which are involved in cell-cell adhesion; (b) Integrins (e.g., Very Late Antigen [VLA]-4 and LFA-1) involved in cell-cell and cell-substratum adhesion; (c) Selectins/LEC-CAMS (cell adhesion molecules with several lectin-like domains) (e.g., Endothelial-Selectin [E-Selectin]) that mediate interaction of leucocytes with endothelial cells; (d) Cadherins, involved in homophilic cell-cell adhesion; and (e) homing receptors that target lymphocytes to specific lymphoid tissues. Other molecules, such as carbohydrate moieties of glycoproteins (e.g., sialyl Lewis X [sLeX] related structures) or constituents of the extracellular matrix (e.g., fibronectin [FN]) may also be involved in adhesion.

In the periphery, different functions and distributions can be attributed to various (endothelial) adhesion molecules, (reviewed by Shimizu et al., 1992), and it is likely that such diversity is also found in the thymus. Two major cell lineages that thymocytes encounter within the thymus are the endothelium (described as the "gatekeeper" regulating lympho-

*Corresponding author.

cyte interactions with tissue) and the epithelium. Therefore, in this paper, by concentrating on adhesion molecules found on peripheral endothelium or epithelium (see Table 1), whose ligands may be found on T cells, we attempt to elucidate the role of adhesion molecules during T-cell development within the thymus. Using immunohistochemical techniques on cryostat sections, we studied the expression of the receptor-ligand pairs LFA-1/ICAM-1 or ICAM-2; CD2/LFA-3; VLA-4/VCAM-1 or fibronectin; and the antigen recognized by HECA 452 (HECA 452-Ag)/E-Selectin on human pediatric thymus. We report a differential distribution of adhesion molecules on stromal cells (epithelium, endothelium, and macrophages) within the thymus, and identify the VCAM-1 positive cell subpopulation as cortical macrophages.

### RESULTS

Single immunoperoxidase staining of various adhesion molecules and their ligands on frozen human thymus sections can be seen in Figs. 1-3. Results of double immunoenzyme staining versus "lineage-specific" markers are summarized in Table 2 and double staining of VCAM-1 versus "lineage-specific" markers can be seen in Fig. 4.

### LFA-1, ICAM-1, and ICAM-2

The anti-LFA-1 antibody used (Table 3) recognizes CD11a, the alpha chain of LFA-1. Our results show that this antibody stains thymocytes throughout the cortex and medulla, with medullary thymocytes staining more strongly (Figs. 1a and 1e). Epithelial cells appear LFA-1-negative (however, when lymphocyte staining is strong, it is difficult to exclude very low levels of staining on nonlymphoid cells). Anti-ICAM-1, on the other hand, reveals a predominantly medullary, nonlymphoid pattern of staining (Figs. 1b and 1f). Double staining experiments show that ICAM-1 can be found at relatively high levels on a subpopulation of medullary and cortical epithelial cells, some macrophages, as well as blood vessels. The two different anti-ICÀM-1 antibodies used, 6.5B5 (not shown) and 8.486, gave similar staining patterns, although 8.486 showed stronger staining, especially of epithelial cells (Fig. 1c). This difference may be attributable to a difference in affinity of the antibodies. ICAM-2 was found to be negative in the thymus (Fig. 1d).

### CD2 and LFA-3

Anti-CD2 stains thymocytes, with epithelial and endothelial cells being CD2-negative (Fig. 2a). The

TABLE 1
Characteristics and Peripheral Distribution of the Adhesion Molecules Studied

| T-cell molecule | Mwt(kD) | Family | Distribution (peripheral) | Counterreceptor | Mwt (kD) | Family | Distribution (peripheral) |
|---|---|---|---|---|---|---|---|
| LFA-1 ($\alpha_L\beta_2$; CD11a/CD18) | 180/95 | INT | Lymphocytes, neutrophils, myelomonocytes | ICAM-1 (CD54) | 76–114 | IG | Activated leucocytes, endothelium, epithelial cells, fibroblasts. |
| | | | | ICAM-2 (CD102) | 55–60 | IG | Constitively expressed on leucocytes and endothelium |
| CD2 (LFA-2; T11; Tp50, Leu 5) | 47–58 | IG | T cells NK cells | LFA-3 (CD58) | 50–77 | INT | Various cell types |
| VLA-4 ($\alpha_4\beta_1$; CD49d/CD29; LPAM-2 [mouse]) | 160/130 | INT | Resting lymphocytes, monocytes, melanoma cells | VCAM-1 (INCAM110; CD106) | 90 — | IG | Activated endothelium |
| | | | | Fibronectin | | | Extracellular matrix |
| HECA 452 Ag (sLeX-related antigens) | | CHO | Some T cells and myelomonocytic cells | E-Selectin (ELAM-1; LECAM-2; CD62E) | 115 | SEL | Activated endothelium |

Abbreviations: Mwt = Molecular weight; INT = integrin; IG = immunoglobulin; CHO = carbohydrate; and SEL = selectin.

TABLE 2
Lineages of Cells Expressing Various Adhesion Molecules as Determined by Double Immunoenzyme Labeling of Human Pediatric Thymus Sections

| Cell subpopulation | ICAM-1 | ICAM-2 | LFA-3 | E-Selectin | VCAM-1 |
|---|---|---|---|---|---|
| Cortical epithelium | + + sp | - | + + | - | (+) |
| Medullary epithelium | + + + sp | - | + + | - | (+) |
| Medullary blood vessels | + | - | + | - | (+)/- |
| Blood vessels in C-M junction | (+) | - | + + | (+) | (+)/- |
| Cortical blood vessels | (+) | - | + + | - | (+)/- |
| Blood vessels in septae | + | - | + | + | (+)/- |
| Medullary macrophages | (+)sp | - | (+)sp | - | - |
| Cortical macrophages | (+)sp | - | (+)sp | - | + + + |
| Medullary lymphocytes[a] | - | - | (+)/+ | - | - |
| Cortical lymphocytes[a] | - | - | (+)/+ | - | - |

[a] As seen by distribution on tissue sections. + = weak (+) = very weak + + = moderate + + + = strong = negative C-M = corticomedullary sp = subpopulation.

TABLE 3
Anti-adhesion Molecule Antibodies Used

| Antibody | Isotype/species | Clone/name |
|---|---|---|
| Anti-LFA-1[a] | IgG1/mouse | MHM-24 |
| Anti-ICAM-1[b] | IgG1/mouse | 6.5B5 |
| | IgG1/mouse | 8.486 |
| Anti-ICAM-2[c] | --/rabbit | 2RAP |
| Anti-CD2[a] | IgG1/mouse | T9-10 |
| Anti-LFA-3[d] | IgG1/mouse | TS2/9.1.4.3 |
| HECA 452[b] | IgM/rat | HECA 452 |
| Anti-E-Selectin[b] | IgG1/mouse | 1.2B6 |
| Anti-VLA-4[e] | IgG1/mouse | HP2/1 |
| Anti-VCAM-1[b] | IgG1/mouse | 1G11 |
| | IgG1/mouse | 1E5 |
| | IgG1/mouse | 6D9 |
| | IgG1/mouse | 3B10 |
| | IgG1/mouse | 1.4C3 |
| Anti-fibronectin[a] | --/rabbit | A245 |

Antibodies were obtained either as monoclonal preparations or as polysera. [a] Dako, High Wycombe, U.K. [b]Generous gifts of D. Haskard, R.P.M.S., London, U.K. [c] Generous gift of W. Makgoba, R.P.M.S., London, U.K. [d] ATCC HB 205, ATCC, Rockville, Maryland [e]Serotec, Oxford, U.K.

results obtained by Watt et al., 1992). It does not appear to be expressed on other cell lineages (Fig. 3a)—a pattern completely different to that given by two of its ligands, VCAM-1 and FN (Figs. 3b and 3c, respectively). Several different anti-VCAM-1 antibodies were used (1G11, 1E5, 6D9, 3B10, and 1.4C3), some of which recognize nonoverlapping epitopes. These all gave a similar staining pattern, strongly staining "patches" in the cortex (e.g. Fig. 3d showing staining obtained with 3B10), whilst weakly staining some epithelial and endothelial cells. Double immunoenzyme staining of anti-VCAM-1 (1G11) versus anti-macrophage (anti-CD68) reveal these "patches" as cortical macrophages (Fig. 4a). Medullary macrophages are VCAM-1-negative (Fig. 4b). Double immunoenzyme staining of anti-VCAM-1 (1G11) versus anti-laminin (used as a marker for blood vessels and basement membrane) shows that VCAM-1-positive cells often appear closely associated with blood vessels (Fig. 4c).

### DISCUSSION

T-cell development occurs under the influence of stromal elements in the thymus, and probably involves adhesion molecules. Little is known about the role of such molecules within the thymus, (reviewed by Patel and Haynes, 1993), although a degree of circumstantial evidence supports their involvement in thymopoiesis.

In many cases, adhesion molecule expression or binding affinity can be increased following cytokine stimulation or cell activation. Further, one adhesion "receptor" may have multiple ligands, or multiple adhesion receptors may exist for one particular ligand. Such potential for upregulation of avidity and expression, the existence of multiple adhesion pathways, and the expression of different adhesion molecules leading to different intracellular events (as suggested by Albelda and Buck, 1990) provide a variety of ways in which cells may interact with one another to give a range of results, and is likely to be of importance in lymphocyte-stromal interactions in the thymus.

In this paper, we have studied the expression of various adhesion molecules on stromal cells, and compared expression to that of their ligands *in situ* using immunohistochemical techniques. It should be noted that some antigens lose their antigenicity during tissue preparation or may be present at levels too low to be detected by the techniques used in this paper, yet may still be of biological significance.

### LFA-1, ICAM-1, and ICAM-2

Our studies show that LFA-1 appears to be expressed on all thymocytes, and at slightly higher-levels in the medulla. This would agree with experiments where LFA-1 is expressed on memory extent on corticomedullary blood vessels, suggesting that it is important in leucocyte traffic. It was noted that neutrophils were commonly found near these E-Selectin positive cells, implying that E-Selectin may be involved in neutrophil recruitment. In the thymus, it is possible that leucocytes produce IL-1 (known to increase E-Selectin synthesis in peripheral endothelium), and thus increase leucocyte interaction with thymic endothelium. E-Selectin may function in initiating leucocyte-endothelium cell adhesion, and hence it may be important in recruitment of T-cell precursors to various thymic subcompartments. If this was the case, one would expect the ligand for E-Selectin to be found on leucocytes/thymocytes. However, in our experiments, expression of the "assumed" ligand (HECA 452-Ag) seems to be localized mainly to blood vessels in the corticomedullary junction, although some staining can be seen on extrathymic blood vessels, neutrophils, and on cells in the medulla. These latter were not identified in these experiments, but two color flow cytometry will indicate if some are T-cell precursors, although Picker et al. (1990) have found that 99% of thymocytes are HECA 452-negative.

Thus, our findings support the involvement of E-Selectin and HECA 452-Ag in leucocyte traffic/endothelial cell interaction rather than in thymocyte development itself.

### VLA-4, VCAM-1, and FN

VLA-4 can bind VCAM-1, or the extracellular matrix molecule FN.

Our experiments show that VLA-4 is expressed on thymocytes (at slightly higher levels in the cortex, which is consistent with flow cytometric data [Watt et al., 1992)]. However, its two ligands show totally different patterns of expression. FN forms part of the extracellular matrix, especially in the medulla — as noted in other mammals. This conserved nature of thymic FN distribution is thought to imply a functional role for extracellular matrix (Utsumi et al., 1991; Savino et al., 1993). We show that VCAM-1 is mainly expressed on cortical macrophages. This suggests that VLA-4 interacts with different ligands at different stages of thymic development. The distribution of VLA-4 is greater than the combined distribution of its ligands. This may imply that there is control of specific interactions by modification of VLA-4 affinity. VCAM-1 may be involved in an interaction between early thymocytes and macrophages via VLA-4 (expressed at higher levels on cortical thymocytes than medullary thymocytes), consistent with data obtained by Kyewski et al., (1982). Moreover, VCAM-1 expression is modulated by the T-cell–produced cytokine IL-4, suggesting that developing T cells may influence their stromal microenvironment (Ritter and Boyd, 1993). We also observed close association of VCAM-$1^+$ macrophages with cortical capillaries, although the developmental significance of this is unclear.

In this paper, we have shown that there is a differential distribution of adhesion molecules within different thymic areas, and that some adhesion molecules such as ICAM-1 are shared by different lineages, whereas others, such as E-Selectin are exclusively found on thymic blood vessels. We have also shown that even where lymphocyte ligands (e.g., LFA-1, CD2, and VLA-4) have a broad distribution on thymocytes, their corresponding adhesion ligands may show more selective distributions, suggesting that different interactions/functions occur in different regions of the thymus. Overall, our results suggest that adhesion molecules are likely to be important in T-cell development, although functional experiments are needed to clarify their role.

### MATERIALS AND METHODS

### Thymus Sections

Thymus samples were obtained from patients (aged 0–4 years) undergoing cardiac surgery. Blocks of tissue were frozen in liquid nitrogen, and 6μm cryostat sections cut. Sections were fixed in acetone and stored at -20°C. Experiments were repeated on several different thymuses.

### Antibodies

All antibodies were titrated before use and subsequently used at the appropriate dilution.

*Primary antibodies* Anti-adhesion molecule antibodies used are outlined in Table 3. "Lineage-specific" markers used included rabbit anti-keratin (Dako, High Wycombe, U.K.) to identify epithelial cells, rabbit anti-laminin (Serotec, Oxford, U.K.) to identify basement membrane associated with capsule and blood vessels, and mouse anti-CD68 (Dako) to identify macrophages.

*Secondary antibodies* Peroxidase conjugated rabbit anti-mouse Ig (Dako); peroxidase conjugated swine anti-rabbit Ig (Dako); .peroxidase conjugated rabbit anti-rat Ig (Dako); alkaline-phosphatase conjugated swine anti-rabbit Ig (Dako); and alkaline-phosphatase conjugated rabbit anti-mouse Ig (Dako). These were all prepared and preincubated with 5% normal human serum/TBS for 15–30 min to block binding to endogenous Ig in sections.

### Immunohistochemistry

*Single immunoenzyme labeling* This labeling determines distribution of adhesion molecules. Sections were incubated in primary antibodies against adhesion molecules, followed by peroxidase conjugated secondary antibody. The reaction was developed using 0.1% 3,3′-diaminobenzidine tetrahydrochloride (DAB) (Sigma, Poole, U.K.) as chromogen substrate with 0.015% $H_2O_2$. Sections were counterstained with haematoxylin and mounted in Kaiser's gelatin-based mountant.

*Double immunoenzyme labeling* This labeling determines lineage of cells stained with anti-ICAM-1, -VCAM-1, -E-Selectin, and -LFA-3. Sections were incubated with these antibodies, followed by peroxidase conjugated secondary antibody, and development of reaction with DAB/$H_2O_2$ substrate. Sections were then incubated with lineage-specific antibodies (anti-keratin, anti-laminin, and anti-macrophage, CD68), followed by alkaline-phosphatase conjugated secondary antibody, and the reaction developed using 0.1% fast blue BB salt (Sigma) with 0.02% napthol AS-MX phosphate (Sigma), 2% dimethyl formamide (BDH, Poole, U.K.), and 0.2% levamisole (Sigma). (Use of immunoenzymatic techniques avoids problems with cross reactivity; antibodies that are used in the peroxidase step lose their antigenicity and binding capacity once this step is developed, and thus do not interfere with the subsequent alkaline phosphatase step.) No counterstaining was carried out. Slides were mounted as before. Sections were photographed using Kodacolor Gold ASA 200 film.

### ACKNOWLEDGMENTS

The authors would like to thank Dr. D. Haskard, and Dr. W. Makgoba for generous gifts of antibodies, and the staff at Great Ormond Street Children's Hospital for collection of thymus samples.

*(Received November 12, 1993)*

*(Accepted January 26, 1994)*

### REFERENCES

Albelda S.M., and Buck C.A. (1990). Integrins and other cell adhesion molecules. FASEB J. **4**: 2868–2880

Arulanandam A.R., Moingeon P., Concino M.F., Recny M.A., Kato K., Koyasa S., and Reinherz L. (1993). A soluble multimetric recombinant CD2 protein identifies CD48 as a low affinity ligand for human CD2: Divergence of CD2 ligânds during the evolution of humans and mice. J. Exp. Med. **177**: 1439–1450.

Berg L.J., Pullen A.M., Fazekas de St. Groth B., Mathis D., Benoist C., and Davis M.M. (1989). Antigen/major histocompatibility complex-specific T cells are preferentially exported from the thymus in the presence of their MHC ligand. Cell **58**: 1035–1046.

Berg E.L., Yoshino T., Rott L.S., Robinson, M.K., Warnock A., Kishimoto T.K., Picker L.J., and Butcher E.C. (1991). The cutaneous lymphocyte antigen is a lymphocyte homing receptor for the vascular lectin endothelial cell–leukocyte adhesion molecule 1. J. Exp. Med. **174**: 1461–1466.

Boyd R.L., and Hugo P. (1991). Towards an integrated view of thymopoiesis. Immunol. Today **12**: 71–79.

Boyd R.L., Tucek C.L., Godfrey D.I., Izon D.J., Wilson T.J., Davidson N.J., Bean A.G.D., Ladyman H.M., Ritter M.A., and Hugo P. (1993). The thymic microenvironment. Immunol. Today **14**: 445–459.

Carlow D.A., van Oers S.C.N., Teh S-J., and Teh H-S (1992). Deletion of antigen-specific immature thymocytes by dendritic cells requires LFA-1/ICAM interactions. J. Immunol. **148**: 1595–1603.

de Fougerolles A.R., and Springer T.A. (1992). Intercellular Adhesion Molecule 3, a third adhesion counter receptor for Lymphocyte Function-associated Molecule 1 on resting lymphocytes. J. Exp. Med. **175**: 185–190.

de Fougerolles A.R., Stacker S.A., Schwarting R., and Springer T.A. (1991). Characterization of ICAM-2 and evidence for a third counter-receptor for LFA-1. J. Exp. Med. **174**: 253–267.

Denning S.M., Tuck D.T., Vollger L.W., Springer T.A., Singer K.H., and Haynes B.F. (1987). Monoclonal antibodies to CD2 and Lymphocyte Function associated Antigen-3 inhibit human thymic epithelial cell-dependent mature thymocyte activation. J. Immunol. **139**: 2573–2578.

Duijvestijn A.M., Horst E., Pals S.T., Rouse B.N., Steere A.C., Picker L.J., Meijer C.J.L.M., and Butcher E.C. (1988). High endothelial differentiation in human lymphoid and inflammatory tissues defined by monoclonal antibody HECA-452. Am. J. Pathol. **130**: 147–155.

Fine J.S., and Kruisbeek A.M. (1991). The role of LFA-1/ICAM-1 interactions during murine lymphocyte development. J. Immunol. **147**: 2852–2859.

Jenkinson E.J., Jhittay P., Kingston R., and Owen J.J.T. (1985). Studies of the role of the thymic environment in the induction of tolerance to MHC antigens. Transplantation **39**: 331–333.

Kappler J.W., Wade T., White J., Kushnir E., Blackman M., Bill J., Roehm N., and Marrack P. (1987). A T cell receptor V$\beta$ segment that imparts reactivity to a class II major histocompatibility complex product. Cell **49**: 263–271.

Killeen N., Stuart S.G., Littman D.R. (1992). Development and function of T cells in mice with a disrupted CD2. EMBO **11**: 4329–4336.

Kyewski B.A., Jenkinson E.J., Kingston R., Altevogt P., Owen M.J., and Owen J.J.T. (1989). The effects of anti-CD2 antibodies on the differentiation of mouse thymocytes. Eur. J. Immunol. **19**: 951–954.

Kyewski B.A., Rouse R.V., and Kaplan H.S. (1982). Thymocyte rosettes: Multicellular complexes of lymphocytes and bone marrow-derived stromal cells in the mouse thymus. Proc. Natl. Acad. Sci. USA **79**: 5646–5650.

Lo D., and Sprent J. (1986). Identity of cells that imprint H-2 restricted T cell specificity in the thymus. Nature **319**: 672–675.

Marlin S.D., and Springer T.A. (1987). Purified Intercellular Adhesion Molecule-1 (ICAM-1) is a ligand for Lymphocyte Function-associated Antigen 1 (LFA-1). Cell **51**: 813–819.

Marrack P., Lo D., Brinster R., Palmiter R., Burkly L., Flavell R.H., and Kappler J. (1988). The effect of thymus environment on T cell development and tolerance. Cell **53**: 627–634.

Patel D.D., and Haynes B.F. (1993). Cell adhesion molecules involved in intrathymic T cell development. Semin. Immunol. **5**: 283–292.

Pigott R. and Power C. (1993). The adhesion molecule facts book (London: Academic Press).

Picker L.J., Michie S.A., Rott L.S., and Butcher E.C. (1990). A unique phenotype of skin-associated lymphocytes in humans. Preferential expression of the HECA-452 epitope by benign and malignant T cells at cutaneous sites. Am. J. Pathol. **136**: 1053–1068.

Poggi A., and Zocchi M.R. (1992). Cultured human thymocytes lacking CD2 and CD11a/CD18 antigens are functional and adhere to endothelial cells via CD56 or CDw49d molecules. Cell. Immunol. **140**: 319–330.

Ritter M.A., and Crispe I.N. (1992). The thymus (Oxford: IRL Press).

Ritter M.A., and Boyd R.L. (1993). Development in the thymus: It takes two to tango. Immunol. Today **14**: 462–469.

Savino W., Villa-Verde D.M.S., and Lannes-Vieira J. (1993). Extracellular matrix proteins in intrathymic T-cell migration and differentiation? Immunol. Today **14**: 158–161.

Shimizu Y., Newman W., Tanaka Y., and Shaw S. (1992). Lymphocyte interactions with endothelial cells. Immunol. Today **13**: 106–112.

Sprent J., von Boehmer H., and Nabholz M. (1975). Association of immunity and tolerance to host H-2 determinants in irradiated F1 hybrid mice reconstituted with bone marrow cells from one parental strain. J. Exp. Med. **142**: 321–331.

Springer, T.A. (1990). Adhesion receptors of the immune system. Nature **346**: 425–434.

Staunton D.E., Dustin M.L., and Springer T.A. (1989). Functional cloning of ICAM-2, a cell adhesion ligand for LFA-1 homologous to ICAM-1. Nature **339**: 61–64.

Utsumi K., Sawada M., Narumiya S., Nagamine J., Sakata T., Iwagami S., Yasumichi K., Teraoka H., Hirano H., Ogata M., Hamaoka T., and Fujiwara H. (1991). Adhesion of immature thymocytes to thymic stromal cells through fibronectin molecules and its significance for the induction of thymocyte differentiation. Proc. Natl. Acad. Sci. USA **88**: 5685–5689.

Vaseux R., Hoffman P.A., Tomita J.K., Dickinson E.S., Jasman R.L., St. John T., and Gallatin W.M. (1992) Cloning and characterization of a new intracellular adhesion molecule ICAM-R. Nature **360**: 485–488.

Watt S.M., Thomas J.A., Edwards A.J., Murdoch S.J., and Horton M.A. (1992). Adhesion receptors are differentially expressed on developing thymocytes and epithelium in human thymus. Exp. Hematol **20**: 1101–11.

Zocchi M.R., Marelli F., and Poggi A. (1990). Simultaneous cytofluorometric analysis for the expression of cytoplasmic antigens and DNA content in $CD3^-$ human thymocytes. Cytometry **11**: 883–887.

